# Draft Genome Sequence of Plant Growth-Promoting Bacillus velezensis BS89

**DOI:** 10.1128/MRA.01294-20

**Published:** 2021-01-07

**Authors:** Vladimir K. Chebotar, Gerben P. Voshol, Natalia V. Malfanova, Elena P. Chizhevskaya, Alexander N. Zaplatkin, Oksana V. Komarova, Maria E. Baganova, Alexander M. Lazarev, Svetlana V. Balakina

**Affiliations:** a All-Russia Research Institute of Agricultural Microbiology, St. Petersburg, Russia; b Institute of Biology Leiden, Leiden, Netherlands; c All-Russian Research Institute of Plant Protection, St. Petersburg, Russia; d Leningrad Research Institute for Applied Agricultural Science “Belogorka,” Leningrad Region, Russia; University of Arizona

## Abstract

The plant growth-promoting bacterium Bacillus velezensis BS89 was isolated from the rhizosphere of winter wheat. Strain BS89 has the ability to promote plant growth and produce a mix of auxins and vitamins. Here, we sequenced the complete genome of this strain to understand the molecular mechanisms underlying its beneficial activities.

## ANNOUNCEMENT

Bacillus velezensis BS89 was originally isolated from the roots of winter wheat plants, variety Lira, using chernozem soil of the Republic of Moldova as described in reference 1. *In vitro* studies aimed at elucidating the potential plant-beneficial metabolites of BS89 showed that this strain produces a mix of auxins, hydrolytic enzymes, and vitamins. The strain was stored in a freezer (Sanyo, Japan) at −80°C as cell suspensions from a single colony in 20% glycerol. We used potato-dextrose agar (PDA; Sigma-Aldrich, USA) plates for passages. Here, we performed whole-genome sequencing of strain BS89 to understand the molecular mechanisms responsible for its beneficial properties.

Strain BS89 was grown in LB medium at 30°C overnight (a single colony was used for plate inoculation). The total cellular DNA was isolated using the cetyltrimethylammonium bromide (CTAB)-NaCl method (2). A DNA library was constructed using the Nextera DNA Flex kit (Illumina, USA) according to the manufacturer’s protocols. Sequencing was performed with an Illumina MiSeq platform using a MiSeq reagent kit V3 with 2 × 300-bp reads.

A total of 1,052,328 paired-end reads with a mean read length of 280 bp were obtained. The Illumina read quality was initially checked using FastQC v0.11.2, and adapter sequences were trimmed using Trimmomatic v0.33 (3). Default parameters were used for all software. The genome was assembled using the A5 assembler v2014-11-20 (4). The analysis described in this paper took place on the Prokka (5) annotation system, while the public genome at NCBI was annotated using PGAP (6). Coding sequences were identified with Prodigal v2.6 (7). rRNA and tRNA genes were located using RNAmmer v1.2 (8) and ARAGORN v1.2.36 (9), respectively. The final assembly contained 3,881,265 bp in 19 contigs with an average G+C content of 46.4%. The maximum contig size was 838,149 bp, and the *N*_50_ value was 428,979 bp. A total of 3,726 protein-coding sequences (CDS), 11 rRNA operons, 84 tRNA genes, and 1 transfer-messenger RNA (tmRNA) were predicted.

Comparison of the obtained complete genome sequences with the GenBank database enabled assignment of the strain to the species *B. velezensis*. The average nucleotide identity (ANI), calculated using ANIcalculator v1.0, between the genome of strain BS89 and that of Bacillus amyloliquefaciens subsp. *plantarum* CC178 (10) was 99.98% (CC178 matches *B. velezensis* in ANI and Genome-to-Genome Distance Calculator [GGDC] analyses). The genome analysis of strain BS89 revealed the presence of gene clusters responsible for the synthesis of plant growth-promoting metabolites (indole-3-acetic acid [IAA] and volatiles), numerous hydrolytic enzymes, vitamins, siderophores, and antimicrobial compounds (surfactin, fengycin, bacilysin, macrolactin, difficidin, bacillaene, and plantazolicin). The complete genome sequence of Bacillus velezensis BS89 will contribute to revealing the molecular mechanisms of plant growth stimulation and should facilitate its applications in agriculture.

### Data availability.

The complete genome sequence was deposited in the National Center for Biotechnology Information (NCBI) under the accession number MPHE00000000. The version described in this paper is the first version (MPHE01000000). The raw sequences have been deposited in the Sequence Read Archive under the accession number SRR12962996.
